# Current Antimicrobial Stewardship Practice and Education in Russian Hospitals: Results of a Multicenter Survey

**DOI:** 10.3390/antibiotics10080892

**Published:** 2021-07-22

**Authors:** Ivan Palagin, Svetlana Rachina, Marina Sukhorukova, Irina Nizhegorodtseva, Ulyana Portnyagina, Svetlana Gordeeva, Elena Burasova, Vladimir Bagin, Olga Domanskaya, Dilip Nathwani, Roman Kozlov

**Affiliations:** 1 Institute of Antimicrobial Chemotherapy, Smolensk State Medical University, 214019 Smolensk, Russia; Marina.Sukhorukova@antibiotic.ru (M.S.); Roman.Kozlov@antibiotic.ru (R.K.); 2 Sechenov First Moscow State Medical University, 119435 Moscow, Russia; Svetlana.Ratchina@antibiotic.ru; 3 State Budgetary Healthcare Institution “Regional Clinical Hospital #2”, The Ministry of Health of Krasnodar Region, 350012 Krasnodar, Russia; nigirin@mail.ru; 4 State Budgetary Institution of the Republic of Sakha (Yakutia) “Regional Hospital #2 Emergency Medical Center”, 677005 Yakutsk, Russia; ulyana-nsk@mail.ru; 5 State Regional Budgetary Healthcare Institution “Murmansk Regional Clinical Hospital n.a. P.A. Bayandin”, 183032 Murmansk, Russia; svetalgor@mail.ru; 6 State Autonomous Healthcare Institution “Republican Clinical Hospital n.a. N.A. Semashko”, The Ministry of Health of the Republic of Buryatia, 670031 Ulan-Ude, Russia; baklab2013@yandex.ru; 7 Medical Association “New Hospital”, 620109 Ekaterinburg, Russia; baginvla@gmail.com; 8 Kuzbas Children’s Clinical Hospital n.a. Professor Y.E. Malakhovskiy, 654063 Novokuznetsk, Russia; olga-domanskaya@mail.ru; 9 Ninewells Hospital and Medical School, Dundee DD1 9SY, Scotland, UK; dilip.nathwani@nhs.net

**Keywords:** antimicrobial stewardship, education, postgraduate training, antibiotics, survey

## Abstract

Proper antibiotic usage education and training of medical students and healthcare professionals is the cornerstone to implement antimicrobial stewardship (AMS) programs worldwide. We conducted this voluntary and anonymous survey on current and preferred educational provision of AMS in Russia. Among 1358 polled respondents from six participating Centers located in geographically remote Federal Districts of Russia, the majority were nurses (52.8%) and doctors (42.0%). Results of the survey demonstrated better coverage of education in AMS on an undergraduate level (57.1%). More than half of respondents in total (52.4%) stated they had not received any postgraduate training. Those 38.4% respondents who received postgraduate teaching in AMS stated that it had been provided substantially by an employing hospital (28.4%) or by a medical university/college (22.3%). According to the conducted survey, the methods of education in AMS in Russian Federation mainly include traditional face-to-face lectures, presentations and provision with clinical guidelines, recommendations and printed materials. The involvement of e-learning and web-based online approaches was lacking. The survey allowed us the identify the key problems associated with training of healthcare workers in this field, in particular the varying availability of under- and postgraduate education in different parts of Russia.

## 1. Introduction

Antimicrobial (AM) prescribing is the key factor contributing to a recognized global threat of antimicrobial resistance (AMR). It is widely accepted that antimicrobial stewardship (AMS) is the crucial strategy to combat AMR. Proper antibiotic usage education and training of medical students and healthcare professionals is the cornerstone to implement AMS programs worldwide [1,2,3,4]. The current situation tends to be more critical and meaningful during the COVID-19 pandemic due to increased inappropriate AM prescribing [5,6]. A significant increase in the prevalence of multiresistant Gram negative bacteria has been observed among inpatients in Russia. According to the AMRmap, there is a universal distribution of nosocomial strains of Enterobacterales producing extended-spectrum beta-lactamases (ESBLs) and carbapenem-resistant *Pseudomonas aeruginosa* and *Acinetobacter baumanii* isolates in the hospital setting that limits options for empirical AB choice [7].

Education in AMS involves many aspects including but not limiting appropriate selection and prescribing antimicrobials, optimizing doses and duration and minimizing toxicity and side-effects so as to optimize clinical, economic and microbiological outcomes such as reducing the occurrence of AMR. This is usually combined with education in robust infection prevention and control practice.

The purpose of this study was to assess:

- the current level of postgraduate educational and training activity amongst healthcare practitioners involved in AMS in Russia;
- the availability of human resources to support AMS;
- the organization of education and training within Russian hospitals;
- the demand for AMS education, including the organization, content and preferred methods of delivery.

In order to combat the growing problem of AMR, the national strategy to prevent the spread of AMR and plan for its implementation were approved by the Russian government in 2017 and 2020, respectively. A number of national guidelines targeting appropriate AM usage have been developed. Multiple AMS regional interventions and campaigns demonstrated a beneficial effect on the practice of AM usage [8,9,10].

The education survey was conducted as a part of the WHO Global Campaign [11] and Russian National Action Plan against AMR [12] in order to inform the development of interventions that will improve effective prescribing with a view to reducing AMR rates.

## 2. Methods and Material

### 2.1. Study Design

A voluntary and anonymous survey on current and preferred educational provision on AMS with a total number of 1358 respondents from 6 participating Centers located in geographically remote Federal Districts (FD) of the Russian Federation (RF) was conducted. The questionnaire was carefully planned and developed jointly by experts in the fields of antimicrobial stewardship education and clinical practice on antimicrobial stewardship from the British Society of Antimicrobial Chemotherapy (BSAC) and the Interregional Association for Clinical Microbiology and Antimicrobial Chemotherapy (IACMAC) with adaptation and translation into Russian (see in Supplementary Materials).

### 2.2. Piloting

To ensure the validity of the included coverage questions, the survey was tested on a pilot sample of 10 respondents from the Smolensk State Regional Hospital—a typical Russian healthcare institution providing specialized treatment for inpatient (overnight) stays. For piloting, the same survey methods (web- or paper-based questionnaire) in equal proportions were tested. After piloting the survey was insignificantly corrected (the wording, order of questions and design of the questionnaire) and the final version circulated.

### 2.3. Questionnaire Layout

The final version of the questionnaire was composed of 35 questions divided into separate sections:

- respondent’s main characteristics
  – geographical work location
  – type of organization
  – medical specialty (clinical pharmacology/microbiology/pharmacy/public health/epidemiology/surgery/therapy/etc.)
  – profession (doctor/pharmacist/nurse/other)
  – role in antimicrobial stewardship (prescription/administration/education/etc.)
- personal education and training in AMS (undergraduate and postgraduate)
- institution/organization main characteristics
  – description of microbiological service
  – AMS team availability
- organization of education and training in AMS in the institution/organization
  – characteristics of educational process
  – organizational frameworks
  – covered topics at induction/during employment
  – methods of education
- demands in AMS (preferred topics, learning methods and needs)

Questions were multiple choice, rating scale or free text (open).

### 2.4. Data Collection and Analysis

Respondents were asked to answer the questions independently online (SurveyMonkey) or written using a printed-out questionnaire; results from the latter method were then entered by the operators to the SurveyMonkey platform. Data were collected on SurveyMonkey, which automatically summarized responses using frequencies and percentages as appropriate.

### 2.5. Ethics

All participants were volunteers without any incentive offer. All responses and data collection were anonymous. The questionnaire was anticipated by a covering letter encouraging respondents to participate and containing brief information on the organizers of the study.

## 3. Results

### 3.1. Geographical Distribution of Participating Centers

Presumably, innovative educational approaches were more likely to originate or be adopted in the main cities and the central part of the RF we ensured that centers more peripheral and distant from these areas were represented within the survey. With this in mind we recruited six participating centers: one each in the Southern FD (Krasnodar, *n* = 637), Northwestern FD (Murmansk, *n* = 191), Far Eastern FD (Yakutsk, *n* = 239) and Ural FD (Ekaterinburg, *n* = 79) and two in the Siberian FD (Novokuznetsk, *n* = 52 and Ulan-Ude, *n* = 160). The total number of respondents was 1358 (Table 1).

### 3.2. Main Characteristics of Survey Respondents

Among 1358 polled respondents, the majority were nurses (52.8%) and doctors (42.0%). Microbiologists and pharmacists represented only about 1% of all participants and in one center (Novokuznetsk) all respondents were doctors. In total, 97.7% of participants in the survey represented public or governmental hospitals. More than 70% of respondents stated their medical specialty as internal medicine, surgery or intensive care medicine—31.2%, 19.9% and 19.2%, respectively. In total clinical pharmacology, pharmacy and epidemiology were stated as less 1% for each discipline.

Through a number of multiple-choice questions, the main prescribing related activities of the participants were explored. The responses were as follows in descending order: prescription (33.2%), monitoring of the need and appropriateness of the antimicrobial during therapy (16.2%), teaching about infection diagnosis and treatment (5.5%), development of antimicrobial prescription policy and guidelines (1.8%) and purchase of antimicrobials (1.3%). In total, 59.3% of all surveyed stated “none of the above”. However, it should be noted that in Novokuznetsk, where respondents were only doctors, 98.1% claimed to have some type of role in prescribing, underlining the major role of doctors in implementing AMS programs in the RF.

All baseline characteristics of survey respondents are summarized in Table 2.

### 3.3. Personal Education and Training in Antimicrobial Stewardship

The survey findings demonstrated that within the RF there was better coverage of education in AMS at an undergraduate level (57.1%) compared to 38.4% receiving any postgraduate training. Those receiving postgraduate training in AMS stated it had been provided substantially by their employing hospital (28.4%) or by a medical university/college (22.3%). Postgraduate respondents also identified additional sources of AMS information as conferences/scientific meetings (12.7%), while the least information was delivered by national/regional governmental agencies or pharmaceutical companies (3.5% and 3.3%, respectively) (Table 3).

### 3.4. Role of Microbiologists and AMS Program Implementation in Institution/Organization

A total of 46.6% of surveyed respondents answered positively to the question of whether microbiologists had a clinical component in their work (ward rounds, prescribing, patient review etc.). Microbiologists’ roles in AMS committees/groups availability (72.5%) and participating in AMS interventions (62.7%) were higher (Table 4). Compared to this, doctors have a significant role in these interventions (81.3%) and pharmacists have a role similar to that of microbiologists (62.1%).

### 3.5. Organization of Antimicrobial Stewardship Teaching at the Institution Level

Almost 63% of surveyed respondents stated that their institutions or organizations had a formal strategy or framework for delivering education in AMS. Answers showed that teaching was mainly provided during employment (57.1%) rather than at induction within 3 months of starting the job (24.0%) (Table 5).

As only a limited part of surveyed respondents could specify the topics included in these educational programs, the results of the survey are presented for all centers cumulatively. Notably, respondents could choose as many options from the provided list of covered topics as necessary. The sequencing of topics in the list is presented in Table 6. The most selected topics both at induction and during employment were “adequate and prompt timing of antimicrobial administration” and “adaptation of necessary infection prevention and control measures”—23% and 38%; 22% and 49%, respectively (Table 6). Our survey indicates that education in AMS is mainly provided in traditional face-to-face lectures and through presentations and provision with clinical guidelines, recommendations and printed materials. Electronic/online or mixed methods approaches to learning appear to be scarcely used.

### 3.6. Personal Preferences for Antimicrobial Stewardship Education/Training

**Figure 1:**
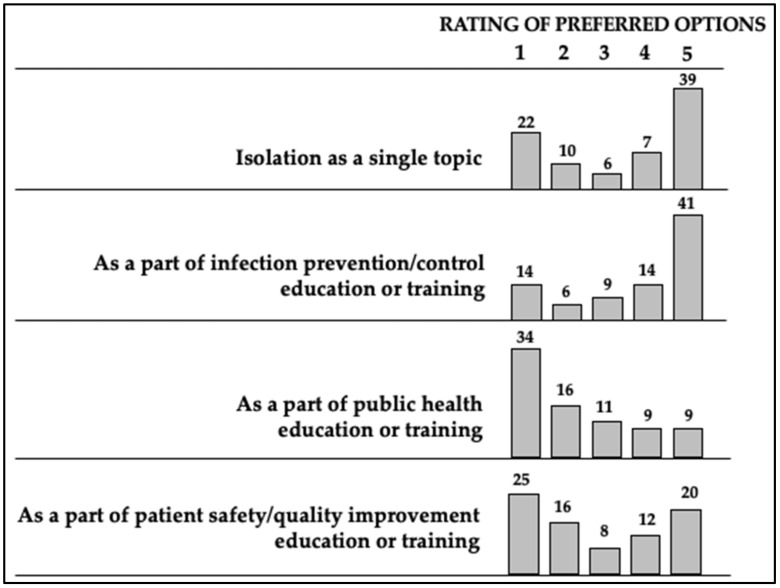
Ratings (1—lowest, 5—highest) for preferred options for antimicrobial stewardship education and training to be delivered. Percentages are depicted above each column, all the remained respondents (up to the total of 100%) did not rate the option during the survey.

Survey participants responded to this section by using a rating score from 1 to 5, with 5 for the most preferred option/statement/topic. The results showed that antimicrobial stewardship education and training is more preferred to be delivered as a part of the infection prevention/control education cycle or as a completely separate cycle/topic (41% and 39% of respondents stated the highest rating, respectively) (Figure 1).

Among listed education topic contents, the most appreciated with the highest rates at the range of 46–56% were:

- Minimization of unnecessary prescribing of antimicrobials
- Adequate and prompt timing of antimicrobial administration
- Appropriate duration of antibiotic treatment and duration as a driver for resistance
- Infection prevention and infection control measures

**Figure 2:**
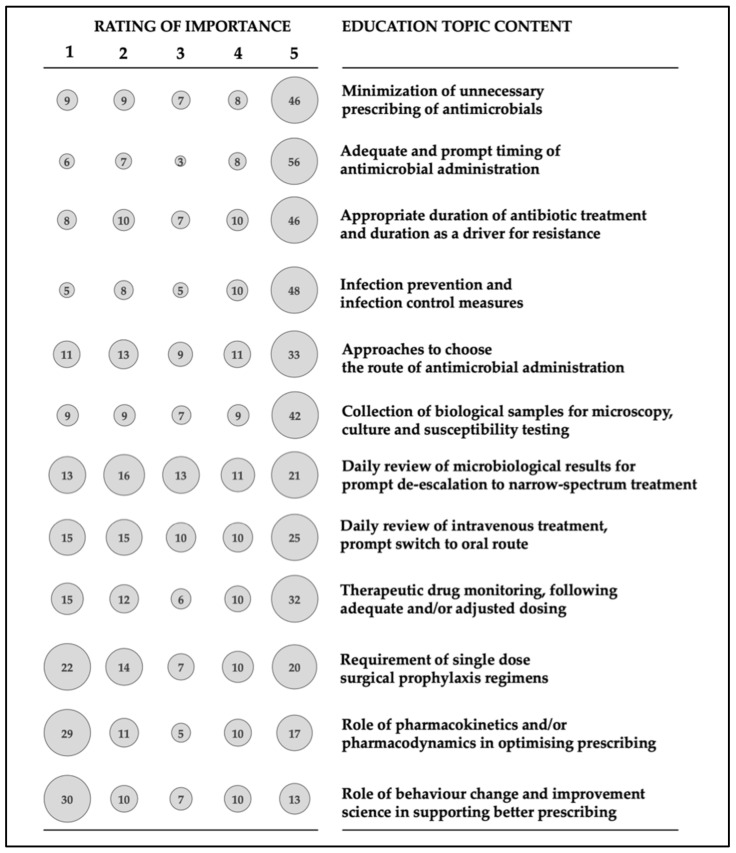
Ratings (1—lowest, 5—highest) for preferred topic content during antimicrobial stewardship education and training. Percentages are depicted above each column; all remaining respondents (up to the total of 100%) did not rate the option during the survey.

Surprisingly, topics such as “Role of pharmacokinetics and/or pharmacodynamics in optimizing prescribing” and “Role of behavior change and improvement science in supporting better prescribing” were quite underrated (29% and 30% of respondents stated the lowest rating, respectively) (Figure 2).

**Figure 3:**
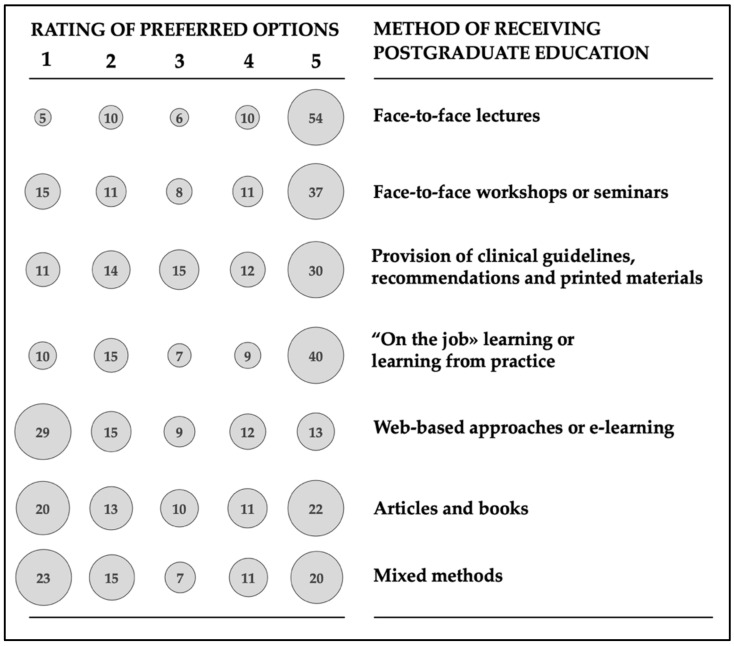
Ratings (1—lowest, 5—highest) for preferred method of receiving postgraduate antimicrobial stewardship education and training. Percentages are depicted above each column, all the remained respondents (up to the total of 100%) did not rate the option during the survey.

Highly preferred methods of receiving postgraduate education on AMS were traditional face-to-face lectures and “on the job” learning/learning from practice (Figure 3). Web-based approaches or e-learning and mixed methods received the highest rating from only 13% and 20% survey participants. This is consistent with data from Table 6.

Most respondents (71%) suggested that the employing hospital and national/regional governmental agency should be responsible for providing proper postgraduate education on AMS—data not shown).

## 4. Discussion

Growing AMR has become a great concern in different countries including Russia [13]. AMS has been recognized as an essential element of national action plans for combating antibiotic-resistant bacteria [11,12,14,15]. Education is a core component of AMS programs. Our survey was conducted in order to recognize the current practice of under- and postgraduate education in AMS in Russia, to identify the most important healthcare professionals in AMS team at the hospital level and to better understand the requirements of healthcare workers in the education. Typically, many such surveys appear to be confined to exploring practice and needs in specialized hospitals in centralized or urban settings as opposed to more remote or rural settings. One of the distinctive features of our study is the focus on including AMS practices across a wide coverage of Russian hospitals settings ranging from the Northwest to the Far East. This broad approach helped us to identify both general patterns and differences in individual regions of the country.

In our survey, apart from those in Krasnodar, the majority of respondents were physicians indicating their main role in AM usage and AMS in the hospital settings. These findings correspond to the results of another study where AMS remained mainly a responsibility of doctors [16].

In the majority of centers enrolled in our survey, specialists in internal medicine, surgery and intensive care medicine prevailed among the respondents. At the same time, participation of pharmacists, clinical pharmacologists and microbiologists was limited with the exception of Murmansk where the proportion of the last two specialties was the highest among the participating centers. A lack of involvement of pharmacists in AMS activities as compared to other developed countries (e.g., UK, USA, Australia) is not surprising. This is due to the peculiarities of the Russian national healthcare system and medical education, where the majority of known responsibilities of pharmacists, such as reviewing of antibiotic (AB) prescriptions, providing a feedback to the prescribers with recommendations for escalation/de-escalation of AB therapy, updating local protocols of AB use, etc., are performed by medically trained clinical pharmacologists [17].

In general, the involvement of microbiologists in the interdisciplinary teamwork is highly welcomed in Russian AMS guidelines [18], but their impact on decision making in the field of AM usage seems to be limited. Only a minority of the respondents mentioned microbiologists involvement in clinical issues. This reflects the current state of microbiological service in Russia, where the “medical microbiologist” clinical specialty in fact was introduced in 2020. In the majority of cases the work of microbiologists is limited to the laboratory, and is aimed, in particular, at pathogen identification and AM susceptibility testing.

There is an overall consistency in understanding the value of continuous under- and postgraduate education in AMS [19,20]. However, it seems that AMS and/or infection control training is not universally provided to healthcare professionals in Russia. In our survey, only 57% of the respondents claimed they received AMS training at the university or in college, and fewer than a half of them had a chance to update it later on in their careers. Considerable heterogeneity in AMS education across regions was also revealed in our study. For example, the highest accessibility was reported in Novokuznetsk and the lowest in Murmansk and Krasnodar. Although most participants reported the availability of the formal framework for education in AMS, which covered different topics (e.g., necessary infection prevention and control measures, adequate and prompt timing of AM administration), a significant number of them could not answer the question on the existence of training in the field of AMS at the hospital level or specify the topics covered during the education. This suggests a poor understanding or awareness of the content of the framework for AMS education.

In our survey, postgraduate education in AMS was usually provided by the employing hospital or medical university/college. Scientific conferences as a source of information were fairly often indicated by participants from Novokuznetsk and Ulan-Ude. Limited access or variability of AMS programs at the national and international level has been demonstrated by different researchers [16,21,22]. For example, Singh S. et al., in qualitative interviews of healthcare professionals from India, highlighted the absence of AMS programs in hospitals, postgraduate education and training for employees [21]. Furthermore, Maraolo A.E. et al. in a recent study demonstrated some substantial differences in organization and training of AMS in 38 European countries [22]. Only 24% of countries responded with the presence of formal national standards for AMS, and these countries reported significant variation between countries in the backgrounds of professionals responsible for AMS, all of which raises concerns about the overall quality of AMS training [22]. These figures are consistent with the delivery of AMS training in Russia. Despite the existence of a framework for AMS education in the majority of settings, this process is not standardized in any way, and the specialists who carry it out do not undergo special training. Nevertheless, according to the results of our survey, microbiologists played an important role in AMS committees and AMS interventions at the hospital level despite a lack of standardized training.

In relation to AMS structures, a relatively high overall percentage of AMS committee/group availability among participating centers (72.5%) as well as availability of interventions targeting AM prescribing (68.5%) appear to be encouraging. This confirms the availability of necessary framework and the presence of some experience in the implementation of AMS initiatives in Russia. This finding supports Russian examples of fairly successful interventions aimed at changing AM use practice both in hospitals and outpatient clinics [23,24]. Davey P. et al. performed a systematic review trying to identify successful interventions targeting AM prescribing to hospital settings [25]. Interestingly, the vast majority of the studies were performed in high income countries of North America or Europe. This clearly raises a question of context as a key factor in implementation and the challenges of extrapolating findings to other regions where conditions for the provision of medical care, culture, resources and potential targets of interventions may differ. For instance, in a recent multisite qualitative study, potential hurdles in implementing of AMS programs in tertiary care settings of Sri Lanka, Kenya and Tanzania were explored [26]. Barriers to improving AM usage included excessively expensive drugs, limited AM availability, reluctance to change current practices regarding AM prescribing and limited diagnostic capabilities.

As for delivery of AMS education, our survey respondents showed no particular preferences. Approximately equal numbers of them were in favor of receiving information within the framework of infection prevention/control education and separate AMS specific education cycles. Minimization of unnecessary prescribing of AM, adequate timing of AB administration and appropriate duration of antibiotic treatment were given the highest priority for further AMS education. Such preferences to some extent reflect inconsistencies identified in assessing the practice of AM use in Russia [27,28]. It should be noted that almost two-thirds of the participants relied for AMS education on the employing hospital, and only a quarter of them considered it their own responsibility (data not shown).

Surprisingly, our respondents preferred traditional education training tools such as face-to-face lectures or workshops and learning from practice more than web-based courses, which are rather popular in other countries [29,30]. Interest in e-learning and web-based online tools in Russia was lacking in general; however, the digitalization of education due to COVID-19 pandemic apparently may change this situation. The availability of a Massive Open Online Course (MOOC) for AMS developed by the University of Dundee and the BSAC [31] in Russian [32] will probably facilitate online learning. Furthermore, attempts at making such courses more bespoke to reflect context are becoming more common [33] and may further enhance uptake of e-learning.

We recognize that our study has some limitations related to both a relatively small number of participating sites and potential sample selection bias. In order to make the data more robust, the survey has to involve more hospitals of different type—primary, tertiary, for specialized medical care. The growing private health care sector in Russia also claims for inclusion in such surveys. The survey was conducted on a voluntary basis, resulting in participation mostly physicians and nurses. Additional efforts should be undertaken to encourage other groups of healthcare professionals as well as authority representatives to participate. Such efforts will help in identifying potential barriers to be faced while implementing AMS initiatives and in making them more efficient. Along with this, to the best of our knowledge, this is the largest study focusing on current state of AMS education in Russian hospitals. This should help inform national, and importantly, local initiatives in developing educational programs and resources.

## 5. Conclusions

Our AMS practice and education survey allowed us the identify the key problems associated with provision of AMS and training of healthcare workers in this field. We highlight varying availability of under- and postgraduate education in different parts of Russia, substantial differences in the framework and content of AMS training programs, preference for work based face to face training provided ideally by the employing hospital or national/regional governmental agency and low involvement of microbiologists and other healthcare professionals (e.g., clinical pharmacologists) regarding antimicrobials, prescribing policy and AMS activities in hospitals. at the hospital level. This information will help inform how AMS services can further evolve and future development of educational frameworks for planning, creating, delivering and ultimately evaluating AMS education at a hospital level in Russia.

## Figures and Tables

**Table 1:** Geographical distribution of participating centers and proportion of respondents.

Federal District	City	*n*	%
Southern	Krasnodar	637	46.9
Northwestern	Murmansk	191	14.1
Far Eastern	Yakutsk	239	17.6
Siberian	Ulan-Ude	160	11.8
	Novokuznetsk	52	3.8
Ural	Ekaterinburg	79	5.8
Total	-	1358	100

**Table 2:** Main characteristics of survey respondents, %.

Characteristics	Krasnodar (*n* = 637)	Murmansk (*n* = 191)	Yakutsk (*n* = 239)	Ulan-Ude (*n* = 160)	Novokuznetsk (*n* = 52)	Ekaterinburg (*n* = 79)	Total (*n* = 1358)
Profession							
Nurse	84.1	37.7	27.2	20.6	-	77.2	52.8
Doctor	15.5	44.5	64.9	73.8	100	13.9	42.0
Microbiologist	0.3	3.1	2.5	0.6	-	-	1.1
Pharmacist	-	0.5	1.7	3.1	-	1.3	0.8
Other	3.3	14.1	3.8	1.9	-	7.6	3.3
Type of organization							
Public or governmental hospital	99.1	95.8	96.2	100	84.6	100	97.7
Primary care center (outpatient department)	-	3.7	2.9	-	15.4	-	1.6
Perinatal/maternity welfare clinic	0.9	-	-	-	-	-	0.4
Private hospital	-	0.5	0.4	-	-	-	0.2
Research institution	-	-	0.4	-	-	-	0.1
Medical specialty							
Internal medicine	37.1	27.8	25.5	33.1	17.3	15.2	31.2
Surgery	8.3	30.9	37.7	31.9	13.5	12.7	19.9
Intensive care medicine	24.0	0.5	13.4	16.3	32.7	39.2	19.2
Obstetrics/gynaecology	23.2	0.5	0.8	0.6	-	1.3	11.2
Microbiology	0.8	15.2	4.6	1.3	-	6.3	3.8
Healthcare administration	-	2.6	4.6	3.8	1.9	3.8	1.9
Clinical pharmacology	0.2	2.1	0.8	1.9	-	-	0.7
Pharmacy	-	-	1.3	3.1	-	1.3	0.7
Epidemiology	-	1.6	0.4	0.6	-	1.3	0.1
Other	6.4	19.4	10.9	7.5	34.6	19.2	11.0
Part of job in relation to antimicrobials (multiple choice answers)							
Prescription	14.8	26.2	49.0	55.0	94.2	67.1	33.2
Monitoring of the need and appropriateness of antimicrobials during therapy	7.7	16.2	24.7	23.8	38.5	29.1	16.2
Teaching about infection diagnosis and treatment	0.3	4.2	6.7	16.3	15.4	17.7	5.5
Development of antimicrobial prescription policy and guidelines	0.6	2.6	2.5	3.8	1.9	2.5	1.8
Purchase	-	0.5	2.5	5.0	1.9	2.5	1.3
None of the above	85.1	56.5	38.5	28.1	1.9	21.5	59.3

**Table 3:** Personal education and training in antimicrobial stewardship, %.

Characteristics	Krasnodar (*n* = 637)	Murmansk (*n* = 191)	Yakutsk (*n* = 239)	Ulan-Ude (*n* = 160)	Novokuznetsk (*n* = 52)	Ekaterinburg (*n* = 79)	Total (*n* = 1358)
Undergraduate education							
Yes	47.9	55.5	69.5	71.9	78.9	54.4	57.1
No	51.0	26.7	14.2	15.0	11.5	20.3	33.6
Not sure	1.1	2.1	11.3	6.9	9.6	17.7	5.0
Not relevant	-	5.8	2.9	2.5	-	3.8	1.8
No answer	-	10.0	2.1	3.8	-	3.8	2.4
Postgraduate education							
Yes	26.5	33.0	47.7	61.3	82.7	43.0	38.4
Provided by employing hospital	19.9	20.9	35.6	50.6	63.5	24.1	28.4
Provided by medical university/college	16.5	13.6	36.4	31.9	32.7	21.5	22.3
Scientific conference	5.2	11.5	15.5	28.1	42.3	17.7	12.7
Provided by professional organization/society	2.4	2.6	5.0	11.9	25.0	3.8	4.9
Provided by another hospital	1.9	1.1	3.4	11.3	5.8	8.9	3.7
Provided by national/regional governmental agency	1.3	1.6	9.2	7.5	1.9	1.3	3.5
Provided by pharmaceutical company	0.6	1.6	4.6	8.1	15.4	7.6	3.3
No	72.2	47.1	36.4	21.9	11.5	41.8	52.4
Not sure	1.1	5.8	13.4	3.1	1.9	11.4	4.8
Not relevant	0.2	4.7	2.1	-	-	2.5	1.3
No answer	-	9.4	0.4	13.8	3.9	1.3	3.2

**Table 4:** Role of microbiologists and antimicrobial stewardship programs implementation in institution/organization, %.

Characteristics	Krasnodar (*n* = 637)	Murmansk (*n* = 191)	Yakutsk (*n* = 239)	Ulan-Ude (*n* = 160)	Novokuznetsk (*n* = 52)	Ekaterinburg (*n* = 79)	Total (*n* = 1358)
Clinical component in work of microbiologist (ward rounds, prescribing etc.)							
Yes	64.5	18.9	40.6	35.0	30.8	21.5	46.6
No	32.0	41.2	37.2	37.5	48.1	60.8	37.2
Not sure	3.5	28.8	20.9	22.5	19.2	16.5	13.7
No answer	-	11.0	1.3	5.0	1.9	1.3	2.5
Antimicrobial stewardship committee/group availability							
Yes	80.4	60.2	62.8	74.4	75.0	63.3	72.5
No	15.7	6.3	6.7	10.6	3.9	8.9	11.3
Not sure	3.9	28.3	29.7	9.4	21.2	25.3	14.4
No answer	-	5.2	0.8	5.6	-	2.5	1.7
Initiatives/interventions targeting antimicrobial prescribing availability							
Yes	79.0	48.2	64.0	64.4	80.8	46.8	68.5
Introduced by doctors	88.7	54.5	78.7	79.4	96.2	88.6	81.3
Introduced by microbiologists	83.7	44.0	50.2	43.8	30.8	36.7	62.7
Introduced by pharmacists	82.3	45.6	47.7	50.0	30.8	27.9	62.1
Introduced by epidemiologists	79.3	61.8	27.2	34.4	32.7	29.1	57.7
Introduced by clinical pharmacologists	-	8.4	20.1	11.9	15.4	3.8	6.9
Introduced by nurses	1.9	7.3	11.3	18.1	7.7	3.8	6.6
No	16.8	7.9	7.5	6.3	3.9	19.0	12.3
Not sure	4.2	31.9	26.8	19.4	11.5	31.7	15.8
No answer	-	12.0	1.7	10.0	3.9	2.5	3.5

**Table 5:** Organization of antimicrobial stewardship teaching at the institution level, %.

Characteristics	Krasnodar (*n* = 637)	Murmansk (*n* = 191)	Yakutsk (*n* = 239)	Ulan-Ude (*n* = 160)	Novokuznetsk (*n* = 52)	Ekaterinburg (*n* = 79)	Total (*n* = 1358)
Formal strategy or framework for delivering education in antimicrobial stewardship							
Yes	76.0	32.5	54.0	71.3	65.4	39.2	62.9
No	19.3	12.6	8.0	3.8	7.7	25.3	14.4
Not sure	4.7	46.6	37.7	16.3	26.9	34.2	20.3
No answer	-	8.4	0.4	8.8	-	1.3	2.4
Institutional education on AMS at induction (within 3 months of starting job)							
Yes	17.6	11.5	23.4	64.4	42.3	13.9	24.0
No	79.6	45.0	35.6	8.1	21.2	59.5	55.2
Not sure	2.8	33.0	39.8	13.1	30.8	22.8	17.0
No answer	-	10.5	1.3	14.4	5.8	3.8	3.8
Institutional education on AMS during employment							
Yes	58.7	35.1	54.0	78.8	88.5	41.8	57.1
No	35.8	15.7	15.9	9.4	-	30.4	24.7
Not sure	5.5	36.1	29.7	6.9	7.7	25.3	15.5
No answer	-	13.1	0.4	5.0	3.9	2.5	2.8

**Table 6:** Specification of teaching in antimicrobial stewardship, %.

	At Induction	During Employment
Covered topics		
Minimize unnecessary prescribing of antimicrobials	18	34
Ensure adequate and prompt timing of antimicrobial administration	23	38
Ensure appropriate duration of antibiotic treatment and duration as a driver for resistance	19	34
Adopt necessary infection prevention and control measures	22	49
Intravenous administration only in severely ill and/or those unable to tolerate oral treatment	13	33
Obtain biological samples for microscopy, culture and sensitivity testing	14	38
Review micro results daily, deescalate to narrow-spectrum treatment promptly	8	16
Review intravenous treatment daily, switch to oral route promptly	8	16
Therapeutic drug monitoring, following adequate and/or adjusted dosing	12	20
Require single dose surgical prophylaxis regimens as appropriate	9	18
The role of pharmacokinetics and/or pharmacodynamics in optimizing prescribing	9	14
The role of behavior change and improvement science in supporting better prescribing	5	10
Methods of education and training		
Face-to-face lectures and presentations	17	55
Face-to-face workshops and seminars	9	29
Providing with clinical guidelines, recommendations and printed materials	17	41
“On the job” learning or learning from practice	17	4
Web-based or e-learning	4	8
Mixed methods	5	9

## Data Availability

Not applicable.

## Supplementary Materials

Questionnaire of the survey is available online at https://www.mdpi.com/article/10.3390/antibiotics10080892/s1.
