# Supplementary material for: Current Antimicrobial Stewardship Practice and Education in Russian Hospitals: Results of a Multicenter Survey
Source: Antibiotics (Basel). 2021 Jul 22;10(8):892. doi: 10.3390/antibiotics10080892 (PMC8388790; doi:10.3390/antibiotics10080892)
Supplement: Supplementary file 1 [file antibiotics-10-00892-s001.zip › antibiotics-1274107-supplementary.pdf]

antibiotics MDPI

Dear Colleague!

We invite you to participate in a scientific project in support of the WHO Global Campaign to Combat Antimicrobial Resistance, led by the Interregional Association for Clinical Microbiology and Antimicrobial Chemotherapy (IACMAC) and the British Society for Antimicrobial Chemotherapy (BSAC). This project is funded by the Fund of the Department of Foreign Affairs and General Welfare of Great Britain and is aimed at implementing the International Strategy for Containing Antimicrobial Resistance in Health Care Facilities in the Russian Federation.

The research is carried out in public and private healthcare facilities, scientific centres with the aim of developing and implementing the most effective measures to improve the rational use of systemic antibiotics (AB) and reduce the level of antibiotic resistance.

Please answer the questions of this questionnaire, which assesses the existing approaches to training in the field of rational use of AB and invites you to choose the most preferable ways from your point of view to improve the educational process in the field of optimizing the use of this group of drugs in your healthcare facility.

WORK DETAILS

1. In which federal district do you work? (Choose from list)
    a. Central
    b. Crimean
    c. Far Eastern
    d. North Caucasian
    e. Siberian
    f. Southern
    g. Ural
    h. Volga
    i. North Western

2. What type of organisation is your main place of work? (Choose from list)
    a. Public or governmental hospital
    b. Private hospital
    c. Outpatient department (primary care center)
    d. Research organization
    e. Perinatal/maternity welfare clinic
    f. Emergency clinic
    g. Non-healthcare related governmental department
    h. Other (please specify)

3. What specialty do you work in? (Choose from list)
    a. Clinical pharmacology
    b. Microbiology
    c. Pharmacy
    d. Public health
    e. Healthcare epidemiology
    f. Surgery
    g. Internal medicine
    h. Intensive care
    i. Obstetrics/gynaecology
    j. Other (please specify)

<u>EDUCATION AND WORKING ACTIVITY</u>

4. What is your profession? (Choose from list)
    a. Doctor
    b. Pharmacist
    c. Nurse
    d. Microbiologist
    e. Other (please specify)

5. As part of your job, do you do any of the following in relation to antimicrobials: (Choose as many as necessary)
    a. Prescribe
    b. Administer
    c. Monitor the need and appropriateness of the antimicrobial during therapy
    d. Teach about infection diagnosis and treatment
    e. Develop antimicrobial prescribing policy and guidelines
    f. None of the above

<u>PERSONAL EDUCATION AND TRAINING</u>

6. Have you received under-graduate education or training in antimicrobial stewardship?
   Yes/ No/ Not relevant as non-clinical/ Not Sure

7. Have you received post-graduate education or training in antimicrobial stewardship?
   Yes/ No/ Not relevant as non-clinical/ Not Sure (if Yes go to Q8, if No go to Q11)

8. Who was the training provided by? Choose multiple options as necessary:
    a. Employing hospital
    b. Another hospital
    c. Medical university or college
    d. Professional scientific society

e. Private healthcare company
f. Pharmaceutical company
g. National or regional governmental agency
h. Scientific conference
i. Other (please specify)

9. Was the training accredited by an external organisation?
   Yes / No/ Not sure (if Yes go to Q10, if No go to Q11)

10. Course Title, accredited by whom (free text)

YOUR INSTITUTION / ORGANISATION

11. In your institution or organisation do medical microbiologists have a clinical component to their work, for example prescribing, patient review, and ward rounds?
    Yes/ No/ Unsure (if Yes or Unsure go to Q 13, if No go to Q12)

12. In your institution or organisation are medical microbiologists based largely in the microbiology laboratory?
    Yes/ No/ Unsure

13. In your institution or organisation is there an infection control/antimicrobial stewardship committee or group?
    Yes/ No/ Unsure

14. Does your institution or organisation have initiatives or interventions that target antimicrobial prescribing?
    Yes/ No/ Not Sure

If Yes, please elaborate ____

15. In your organisation what groups of healthcare professionals are identified as having a role in antimicrobial stewardship? Tick all that apply
    a. Nurses
    b. Pharmacists
    c. Doctors
    d. Microbiologists
    e. Epidemiologists
    f. Other (Please specify) ____

ORGANISATIONAL EDUCATION AND TRAINING

16. In your institution or organisation is there a formal strategy or framework for developing and delivering education and training in antimicrobial stewardship and/or infection control?
Yes/ No/ Unsure

17. Do healthcare workers at your institution or organisation receive education or training in antimicrobial stewardship and/or infection control **at induction** (within three months of starting their job)?
Yes/ No /Unsure (if Yes go to Q18, if No or Unsure go to Q20)

18. Which of the following topics are covered in antimicrobial stewardship and/or infection control education **at induction** (within 3 months of starting the job)? (Choose as many options as necessary)
    a. Minimise unnecessary prescribing of antimicrobials
    b. Ensure adequate and prompt timing of antimicrobial administration
    c. Ensure appropriate duration of antibiotic treatment and duration as a driver for resistance
    d. Adopt necessary infection prevention and control measures
    e. Intravenous administration only in severely ill and/or unable to tolerate oral treatment
    f. Obtain biological samples for microscopy, culture and sensitivity testing
    g. Review micro results daily [assuming access to laboratory results], deescalate to narrow-spectrum treatment promptly
    h. Review intravenous treatment daily, switch to oral route promptly
    i. Therapeutic drug monitoring, following adequate and/or adjusted dosing
    j. Require single dose surgical prophylaxis regimens as appropriate
    k. The role of pharmacokinetics and/or pharmacodynamics in optimising prescribing.
    l. The role of behaviour change and improvement science in supporting better prescribing
    m. Others (please specify) ____

19. What methods for education and training in antibiotic stewardship and/or infection control are used **at induction** (within 3 months of starting their job)? (Choose any many options as necessary)
    a. Face-to-face lectures/presentation
    b. Face-to-face workshops or seminars
    c. Work-placed teaching e.g. workbooks or portfolios of evidence
    d. ‘On the job’ learning or learning from practice
    e. Web-based or e-learning
    f. Mixed methods e.g. e-learning and lectures
    g. Don’t educate
    h. Don’t know

i. Other (please specify) ____

20. Do healthcare workers at your institution or organisation receive education and training on antimicrobials stewardship **throughout their employment**?

    Yes/ No/ Not sure (if Yes go to Q21, if No or Unsure go to Q27)

21. Which of the following topics are covered in antimicrobial stewardship and/or infection control education **during employment**? (Choose as many options as necessary)
    a. Minimise unnecessary prescribing of antimicrobials
    b. Ensure adequate and prompt timing of antimicrobial administration
    c. Ensure appropriate duration of antibiotic treatment and duration as a driver for resistance
    d. Adopt necessary infection prevention and control measures
    e. Intravenous administration only in severely ill and/or unable to tolerate oral treatment
    f. Obtain biological samples for microscopy, culture and sensitivity testing
    g. Review micro results daily [assuming access to laboratory results], deescalate to narrow-spectrum treatment promptly
    h. Review intravenous treatment daily, switch to oral route promptly
    i. Therapeutic drug monitoring, following adequate and/or adjusted dosing
    j. Require single dose surgical prophylaxis regimens as appropriate
    k. The role of pharmacokinetics and/or pharmacodynamics in optimising prescribing.
    l. The role of behaviour change and improvement science in supporting better prescribing
    m. Others (please specify) ____

22. What methods of antimicrobial stewardship education or training are for used **throughout employment**? (Choose as many options as necessary)
    a. Face-to-face lectures/presentations
    b. Face-to-face workshops or seminars
    c. Work-placed teaching e.g. workbooks or portfolios of evidence
    d. ‘On the job’ learning or learning from practice
    e. Web-based or e-learning
    f. Mixed methods e.g. e-learning and lectures
    g. Don’t educate
    h. Don’t know
    i. Other (please specify) ____

23. How frequently is this education or training provided in your institution?
    a. Annually or more frequently
    b. Every one to two years
    c. Every two to five years
    d. Ad hoc

e. Other (please specify) ____

24. Is the education or training that is provided **at induction** mandatory?
Yes/ No/ Not Sure/ Not applicable

25. Is the education or training that is provided **during employment** mandatory?
Yes/ No/ Not Sure/ Not applicable

26. Is attendance at this education or training formally recorded?
Yes/ No/ Not Sure/ Not applicable

27. Which of the following do you think are most important when providing education and training on antimicrobial prescribing? **(Scale of 1-5 with 5 having the greatest importance)**
    a. Minimise unnecessary prescribing of antimicrobials
    b. Ensure adequate and prompt timing of antimicrobial administration
    c. Ensure appropriate duration of antibiotic treatment and duration as a driver for resistance
    d. Adopt necessary infection prevention and control measures
    e. Intravenous administration only in severely ill and/or unable to tolerate oral treatment
    f. Obtain biological samples for microscopy, culture and sensitivity testing
    g. Review micro results daily [assuming access to laboratory results], deescalate to narrow-spectrum treatment promptly;
    h. Review intravenous treatment daily, switch to oral route promptly
    i. Therapeutic drug monitoring, following adequate and/or adjusted dosing
    j. Require single dose surgical prophylaxis regimens as appropriate
    k. The role of pharmacokinetics and/or pharmacodynamics in optimising prescribing.
    l. The role of behaviour change and improvement science in supporting better prescribing

28. Rate the following statements on a scale of 1-5 with 5 being your preferred option.
I would prefer antimicrobial stewardship education and training to be delivered in:
    a. Isolation as a single topic
    b. As part of infection prevention/control education or training
    c. As part of public health education or training
    d. As part of patient safety/quality improvement education or training

LEARNING METHODS AND NEEDS

29. What are your preferred methods of receiving post-graduate clinical education and training in antimicrobial stewardship and prescribing? Scale of 1-5 with 5 your preferred method.
    a. Face-to-face lectures
    b. Face-to-face workshops or seminars
    c. Work-placed teaching e.g. workbooks or portfolios of evidence
    d. 'On the job' learning or learning from practice
    e. Web-based or e-learning
    f. Articles and books
    g. Mixed methods e.g face-to-face lectures and web-based/ e-learning

30. Who do you think should be responsible for providing this education and training? Scale of 1-5 with 5 having the greatest responsibility.
    a. Employing hospital
    b. Employees own responsibility/commitment to be up to date
    c. Another hospital
    d. University or college
    e. Professional organisations e.g. professional society
    f. Private healthcare company
    g. Pharmaceutical industry - e.g drug or diagnostic company
    h. National or regional governmental agency
    i. Conference organisers
    j. Other (please specify)

    ____

31. Do you think measuring staff participation in education activity should be a metric/indicator for antimicrobial stewardship?

    Yes/ No/ Unsure

32. Are you aware of the global free open online course on antimicrobial stewardship available at the following site [https://www.futurelearn.com/courses/antimicrobial-stewardship]:

    Yes/ No/ Unsure

33. Have you participated in the global free on-line course an antimicrobial stewardship available at the following site [https://www.futurelearn.com/courses/antimicrobial-stewardship]:

    Yes/ No/ Unsure

34. Would translation of this educational course into Russian increase uptake/participation in the course?

    Yes/ No/ Unsure

35. Please add up to 2 key observations or comments here in relation to the development, delivery or evaluation of education/training in relation to stewardship.

Thank you for participating in this survey,
your time and involvement are most appreciated!
